# Local Alignment of DNA Sequence Based on Deep Reinforcement Learning

**DOI:** 10.1109/OJEMB.2021.3076156

**Published:** 2021-04-27

**Authors:** Yong-Joon Song, Dong-Ho Cho

**Affiliations:** School of Electrical EngineeringKorea Advanced Institute of Science and Technology 34968 Daejeon 305-701 South Korea

**Keywords:** Deep reinforcement learning, local alignment, meta learning, sequence alignment, sequence comparison

## Abstract

*Goal:* Over the decades, there have been improvements in the sequence alignment algorithm, with significant advances in various aspects such as complexity and accuracy. However, human-defined algorithms have an explicit limitation in view of developmental completeness. This paper introduces a novel local alignment method to obtain optimal sequence alignment based on reinforcement learning. *Methods:* There is a DQNalign algorithm that learns and performs sequence alignment through deep reinforcement learning. This paper proposes a DQN x-drop algorithm that performs local alignment without human intervention by combining the x-drop algorithm with this DQNalign algorithm. The proposed algorithm performs local alignment by repeatedly observing the subsequences and selecting the next alignment direction until the x-drop algorithm terminates the DQNalign algorithm. This proposed algorithm has an advantage in view of linear computational complexity compared to conventional local alignment algorithms. *Results:* This paper compares alignment performance (coverage and identity) and complexity for a fair comparison between the proposed DQN x-drop algorithm and the conventional greedy x-drop algorithm. Firstly, we prove the proposed algorithm's superiority by comparing the two algorithms’ computational complexity through numerical analysis. After that, we tested the alignment performance actual HEV and E.coli sequence datasets. The proposed method shows the comparable identity and coverage performance to the conventional alignment method while having linear complexity for the $X$ parameter. *Conclusions:* Through this study, it was possible to confirm the possibility of a new local alignment algorithm that minimizes computational complexity without human intervention.

## I. Introduction

Sequence alignment is one of the popular methods for analyzing the relationship between biological information by finding similarities between various biological sequence data such as DNA, RNA, and protein. In the early stage of sequencing technology, the dynamic programming-based conventional alignment method was sufficient to analyze the sequences with few nucleotides [1], [2]. However, with the advancement of NGS technology, biological sequence information is increased to the range of millions to billions of base pairs [3]. This advancement in sequence lengths naturally brings the development of sequence alignment methods.

There are many types of the sequence alignment methods depending on the application, such as sequencing sequences with semi-global alignments in NGS, as well as comparing sequences with global, local, and multiple sequence alignments. In the development of methodologies such as global alignment, semi-global alignment, local alignment, and multiple sequence alignment, various heuristic sequence alignment methods were studied to improve the sequence alignment's performance [8]–[16]. Although there have been studies on sequence alignment in various directions, it is hard to develop a sequence alignment method with linear complexity and high alignment performance.

Recently, there have been several efforts to solve the problems of conventional alignment methods. The greedy x-drop algorithm is one of the famous algorithms among the local alignment methods. The greedy x-drop algorithm is an alignment method that terminates the expansion process if the difference between current alignment score and the best alignment score is larger than X. Since conventional algorithm has a complexity linearly proportional to the alignment's length, the complexity problem seems to be solved. However, this greedy x-drop algorithm also has a complexity proportional to a square of the given $X$ parameter. To align sequences in case of a large $X$ parameter, the greedy x-drop algorithm needs a massive number of alignment steps proportional to $O(X^2)$. Therefore, in this paper, we would like to use the DQNalign algorithm, which aligns a sequence pair with linear-complexity and shows satisfactory performance using the deep reinforcement learning method [18].

Up to now, many attempts have recently been made to apply deep learning into sequence comparison researches. At first, there were methods to analyze the result of sequence alignment to classify the sequences or to predict protein structure or analyze the characteristics of sequences with alignment-free features [30]–[34]. These methods use regression or classification methods based on deep learning to predict similarity values resulting from sequence alignment or to predict characteristics of organisms. However, these methods did not directly align sequences and indicated results using the network which is trained from the results of the conventional sequence alignment methods. Moreover, several researchers tried to incorporate deep learning into the sequence alignment process. For instance, there were alignment methods that selected permutation of sequence alignment in progressive multiple sequence alignment [35] or used the entire sequences as the input of the deep neural networks [36]. However, the conventional deep learning-based sequence alignment approaches could not find an appropriate protocol performing alignment for sequences with various sizes. That is, deep learning based sequence alignment methods could be applied only to align for very short sequences of tens to hundreds base pairs because of its fixed input size and network size limitation.

In the previous paper, the authors proposed a method called DQNalign that repeats selecting the next alignment direction while sliding a window using a deep reinforcement learning method. Here, the deep reinforcement learning method is one of the deep learning methods that the deep neural network-based agent select optimal actions to get the maximal total reward in a given environment [19]–[21]. From video games to healthcare, the deep reinforcement learning method can be adopted in various fields. In a previous paper, the authors proposed state, action, and reward appropriate for the sequence alignment system to fit the sequence alignment system into reinforcement learning protocol. This DQNalign method opened the possibility of a completely linear sequence alignment method.

In this paper, we tried to examine the adaptability of this alignment method called DQNalign to various sequence alignments. The DQNalign algorithm, which performs alignment through window sliding, is similar to the gapped extension algorithm in the conventional local alignment method. Therefore, we tried to combine DQNalign with the local alignment method to compare two long complete genomes. So, we developed a novel local alignment algorithm by combining the x-drop algorithm with the DQNalign algorithm. The proposed DQN x-drop algorithm has the advantage of an utterly linear alignment method. Based on this advantage, the proposed algorithm can perform a broader search with low complexity than the conventional greedy x-drop algorithm.

**Fig. 1.**
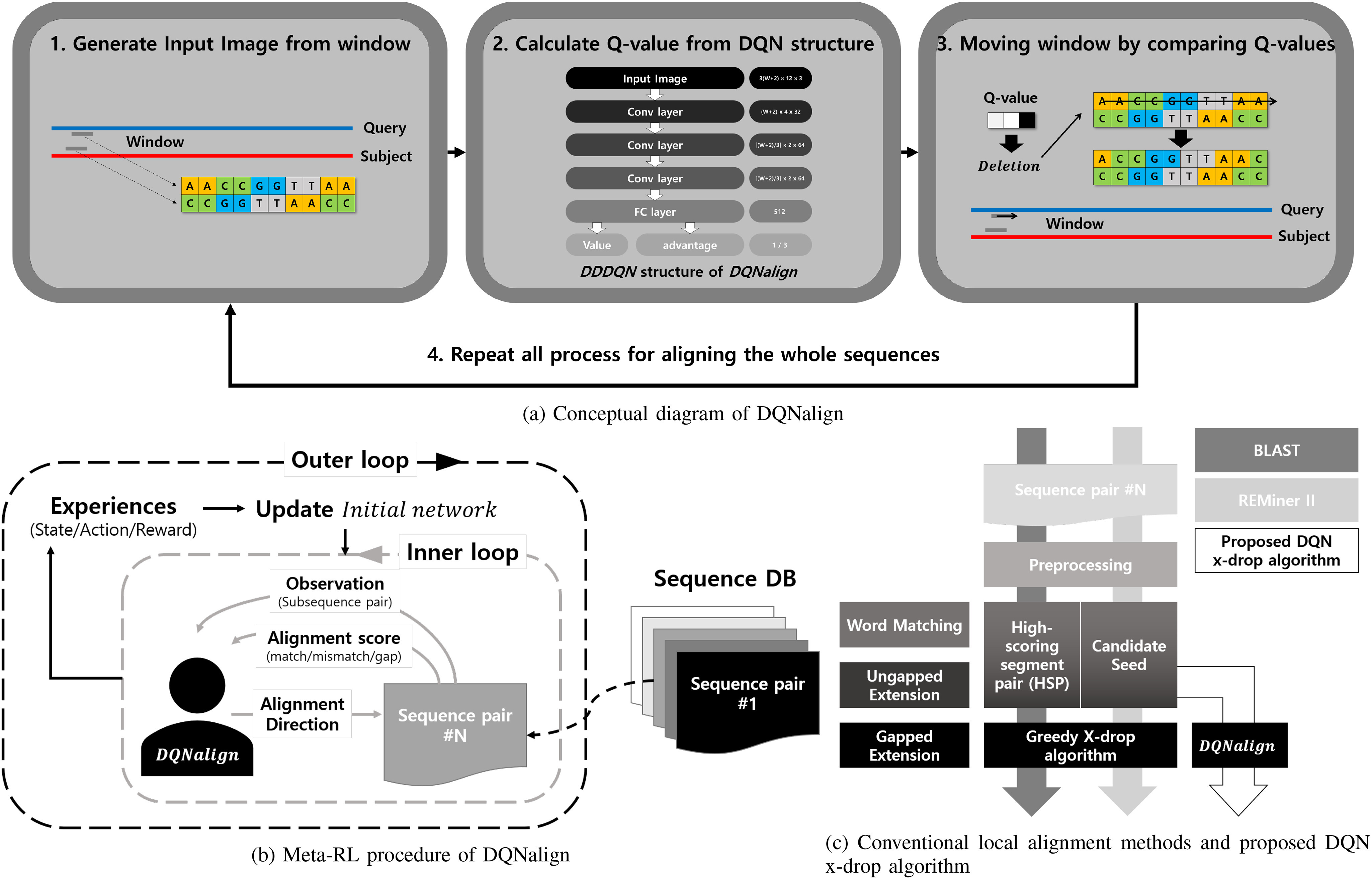
Overview of proposed DQN x-drop algorithm

This paper deals with a new local alignment method based on deep reinforcement learning: In section II, we briefly introduce the conventional greedy x-drop algorithm and discuss how the proposed algorithm differs from the conventional algorithm. In section III, we try to find out how the proposed algorithm exhibits superiority in view of complexity. Therefore, we show how the proposed algorithm can align genome sequences properly with linear complexity, and we compare the alignment performance of the proposed algorithm with that of conventional algorithm to verify and demonstrate the proposed algorithm's superiority in real genome sequences. Finally, we describe the conclusion of this paper and further research subjects in section V.

## II. Materials and Methods

In this paper, we will propose a novel type of the local alignment by combining DQNalign with x-drop algorithm. The detailed explanation and the code implementation of the proposed DQN x-drop algorithm are available at https://github.com/syjqkrtk/DQNalign.

### A. Conventional greedy x-drop algorithm

We will briefly introduce the conventional greedy x-drop algorithm. A greedy x-drop algorithm is a method used in various sequence alignment methods and is a method used to extend the gapped alignment. First, the x-drop algorithm is a method of terminating the alignment when the alignment score falls below the highest score observed so far [6]. This algorithm has advantages, in view of reducing computation time and preventing two distant exact matches from being linked [7].

In particular, the greedy x-drop algorithm is a method that combines dynamic programming-based optimal alignment and x-drop algorithm. Alignment scores are calculated based on the Smith-Waterman algorithm, and the alignment ends when the score falls by more than $X$ in each column. So, this method searches for a region proportional to the square of $X$. Thus, it has a complexity proportional to the square of $X$, and we cannot use a large number of $X$ in conventional greedy x-drop algorithm.

In this paper, BLAST is used as a representative tool of the greedy x-drop algorithm. BLAST is an algorithm that is considered one of the state-of-the-art tools for searching local alignments [8].We used the parameters of megablast for performance comparison of BLAST. To show the performance difference between the conventional greedy x-drop algorithm and the proposed DQN x-drop algorithm, we changed some of the parameters in BLAST as shown in Table S2.

### B. Proposed DQN x-drop algorithm

#### 1) Quick review of DQNalign

Before explaining the proposed DQN x-drop algorithm, we will briefly describe the DQNalign algorithm. DQNalign is an algorithm for aligning sequence pairs using a deep reinforcement learning algorithm [18]. The learned Deep Q-network (DQN) observes only parts of predetermined length (window size) of sequences and continuously selects the optimal alignment direction to proceed. A simple conceptual diagram of the DQNalign algorithm is shown in Fig. 1(a). As shown in the figure, the DQNalign method only needs to determine the next direction immediately from the current position. The DQNalign algorithm consistently showed high performance, irrespective of the sequence pair's identity, by learning which path is optimal in the sequence without any human intervention [18].

To enable reinforcement learning in sequence alignment system, we defined state, action, and reward as follows: sub-sequence pair, alignment direction (forward, deletion, insertion), and alignment scoring system (match, mismatch, gap scores). First, we defined a sub-sequence pair of window sizes as a state in two sequence pairs. And the alignment directions are treated as actions. Then, we can get the changed sequence alignment score as a reward which is decided by selecting alignment directions in the current sub-sequence pair. By defining state, action, and reward, we tried to predict the sum of future rewards for the next alignment direction in a given sub-sequence pair and to advance in the optimal direction through this reinforcement learning system. In this reinforcement learning system, we used the Dueling Double DQN which is one of representative methods of deep reinforcement learning. In detail, Dueling Double DQN uses two different techniques: Dueling DQN and Double DQN. Dueling DQN separates the Q-value into two values (value and advantage). Each value represents the expected sum of future rewards of state and actions. Dueling DQN prevents instability in the learning process. Besides, Double DQN method uses two similar network named main network and target network. The target network slowly approaches to the main network. And, in the training procedure, the action is determined by the Q-values calculated by the target network. Double DQN enables stable selection in the training procedure.

Moreover, we used the $\epsilon$-greedy exploration method to prevent overfitting by randomly experiencing various states and actions during the training. Also, we used the experience buffer which trains the network from the records including state, action, and reward.

Here, we introduce two network structures optimized in the sequence alignment system: DDDQN and faster DDDQN. The detailed DQN structure is depicted in Fig. S1 and Fig. S2. Each network is focused on performance and speed, and in this paper, we used a faster DDDQN structure considering speed to focus the advantage of our algorithm. In detail, DDDQN structure only used the existing methods described above. But,in the case of faster DDDQN, the separable convolutional layer is used which has same the perspective field compared to conventional convolutional layer, but the complexity is greatly reduced. With faster DDDQN structure, we were able to reduce the operations about 1/9 to 1/26 times compared to the DDDQN structure.

#### 2) Proposed meta learning based training procedure

In the early DQNalign, the alignment agent was trained in the environment of virtual sequence sets created by the JC69 model [26]. However, in this paper, a model-agnostic meta-learning (MAML) method is used in consideration of the properties of the actual sequence [23]. The MAML is a kind of meta-learning approach, which can be applied to all learning models using gradient descent. As shown in Fig. 1(b) and Algorithm 1, in the inner loop, the neural network is trained in an environment considering various SNP and indel probability parameters shown in Table S4. The outer loop then updates the final network by evaluating the multiple networks learned in the inner loop. Finally, we fine-tuned the final network of DQNalign using a part of the real genome sequence DB to optimize the neural network in real genome cases [28], [29].

We set the environment distribution with various SNP, indel probability, and maximum indel length with uniform distribution within the range shown in Table S4. In each step of the outer loop, one environment is determined from this environment distribution. In the inner loop, two sequences are generated from the environment. Then, the agent is trained by a general DQN process, and the trained network is tested against other sequences created in the same environment. All the state, action and reward are recorded in the episode buffer. Finally, the initial network in Fig. 1 is updated in the outer loop using these episodes buffer. With this meta training process, we can optimize the DQNalign network for various environments. Then, in test scenarios, we finetune this converged network by using a part of the given sequence DB. Through this process, we were able to confirm the improvement of the alignment performance in the HEV sequence DB as shown in Fig. S3.

Algorithm 1: **MAML Based Training Procedure.**
1: **Inputs**:
$S_{SNP} \gets$ Set of occurrence probability of SNP
$S_{indel} \gets$ Set of occurrence probability of indel
$S_{maxI} \gets$ Set of maximum length of indel
2: **Initialize**:
Initialize a network $DQN_W^0()$
Initialize a replay memory in outer loop $D^{outer}$
3: **for** $outer = 0,M-1$ **do**
4: Pick $P_{SNP} \in S_{SNP}$, $P_{indel} \in S_{indel}$, $maxI \in S_{maxI}$
5: Set $Env \gets (P_{SNP},P_{indel},maxI)$
6: Generate two random sequence $S_1$ and $S^{\prime }_1$
7: Mutate $S_1$ and $S^{\prime }_1$ by $Env$ to get $S_2$ and $S^{\prime }_2$
8: Initialize a replay memory in inner loop $D^{\prime inner}$
9: Copy $DQN_W^{outer}()$ into $DQN_W^{^{\prime }0}()$
10:
$(x,y) \gets (0,0)$

11: **for** $inner = 0,N-1$ **do**
12:
$s_1^{inner} \gets S_1[x:x+W]$

13:
$s_2^{inner} \gets S_2[y:y+W]$

14:
$(Q_{for},Q_{ins},Q_{del}) \gets DQN_W^{^{\prime }inner}(s_1,s_2)$

15:
$a^{inner} \gets argmax(Q_{for},Q_{ins},Q_{del})$

16: Move position $(x,y)$ by a given action $a^{inner}$
17: Get a reward $r^{inner}$ from given action and state
18: Append $(s^{inner},a^{inner},r^{inner},s^{inner+1})$ to $D^{^{\prime }inner}$
19: Update network $DQN_W^{^{\prime }inner+1}()$ with $D^{^{\prime }inner}$
20: **end for**
21: Test network $DQN_W^{^{\prime }N}()$ in new environment $S^{\prime }_1$ and $S^{\prime }_2$
22: Append new trajectories to $D^{outer}$
23: Update network $DQN_W^{outer+1}()$ with $D^{outer}$
24: **end for**
25: **return** $DQN_W^{M}()$

#### 3) Proposed DQN x-drop algorithm

In order to perform local alignment using DQNalign, the x-drop algorithm was applied to the DQNalign algorithm. In Algorithm 2, we have described in detail the proposed DQN x-drop algorithm. Here, the $DQN_W()$ is a deep neural network that receives two subsequences of a given window size as an input and sends out $Q_{for}$, $Q_{ins}$ and $Q_{del}$, which are expected sum of future rewards in the direction of forward, insertion, and deletion. Then, we select the direction with the highest value among the Q values and proceed the alignment process by repeating this DQNalign procedure. Here, the x-drop algorithm terminates the DQNalign procedure if the difference between current alignment score and the best alignment score is larger than X. Then, we can complete the gapped extension process by performing the proposed DQN x-drop algorithm in the upstream and downstream directions. To compare the proposed algorithm with the conventional algorithm, we use the preprocessing and seeding procedure of the REMiner II method to reduce the candidate seeds with n-hit method [9], as shown in Fig. 1(c). The detailed parameters of REMiner II are given in Table S1.

**Fig. 2.**
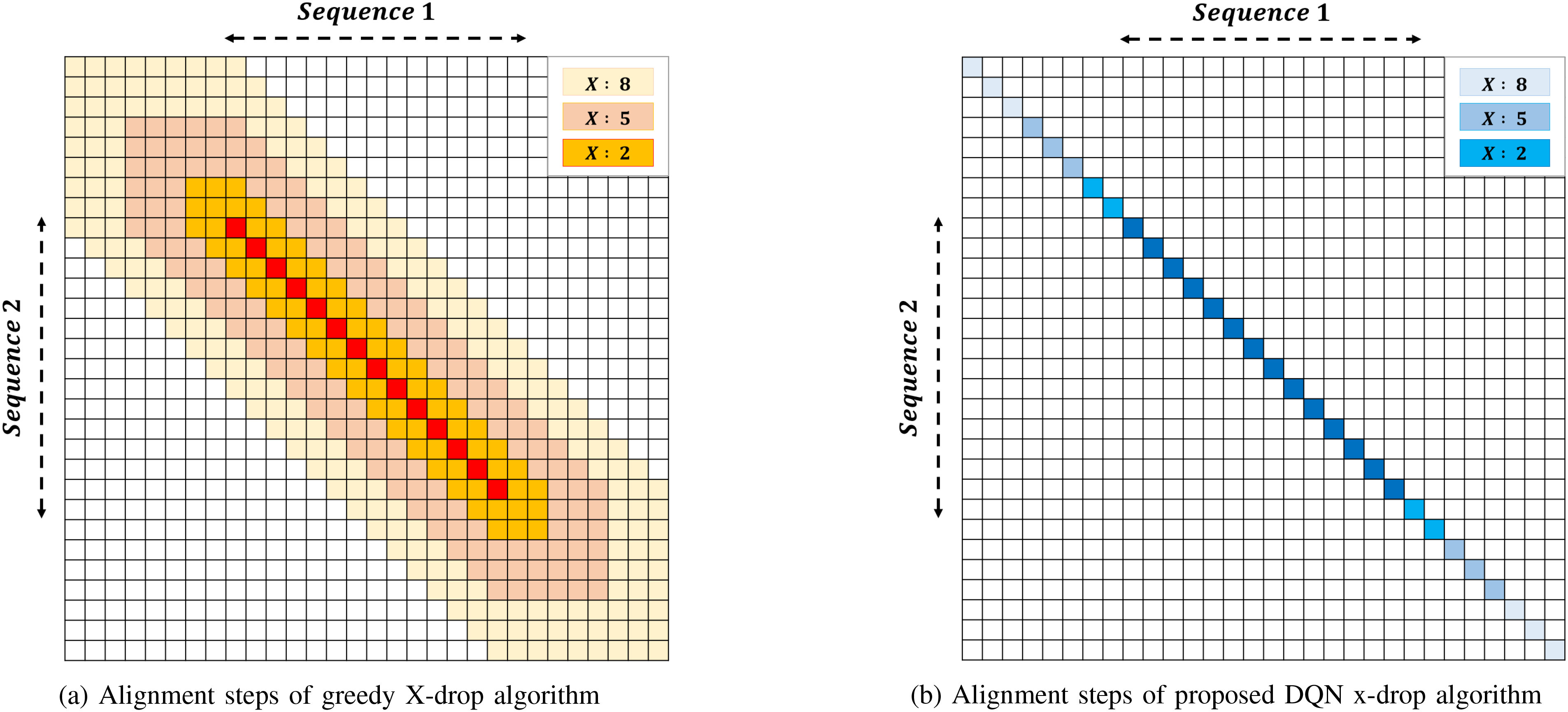
Alignment step comparison of two sequence alignment methods

Algorithm 2: **Proposed DQN X-Drop Algorithm.**
1: **Inputs**:
$S_1 \gets$ Query sequence
$S_2 \gets$ Subject sequence
$(x,y) \gets$ Start position of a given candidate seed
2: **Initialize**:
$DQN_W^M() \gets$ Converged network of window size $W$
$X \gets$ x-drop parameter

$Score \gets 0$

$Best \gets 0$

$Path \gets \emptyset$

$Align \gets \emptyset$

3: **while** $Score > Best - X$ **do**
4:
$s_1 \gets S_1[x:x+W]$

5:
$s_2 \gets S_2[y:y+W]$

6:
$(Q_{for},Q_{ins},Q_{del}) \gets DQN_W(s_1,s_2)$

7:
$a \gets argmax(Q_{for},Q_{ins},Q_{del})$

8: Move position $(x,y)$ by a given action $a$
9: Append $(S_1[x],S_2[y])$ to $Path$
10: Get a reward $r$ from given $a$ and $(S_1[x],S_2[y])$
11:
$Score \gets Score +r$

12: **if** $Score > Best$ **then**
13:
$Best \gets Score$

14:
$Align \gets Path$

15: **end if**
16: **end while**
17: **return** $Align$

**Fig. 3.**
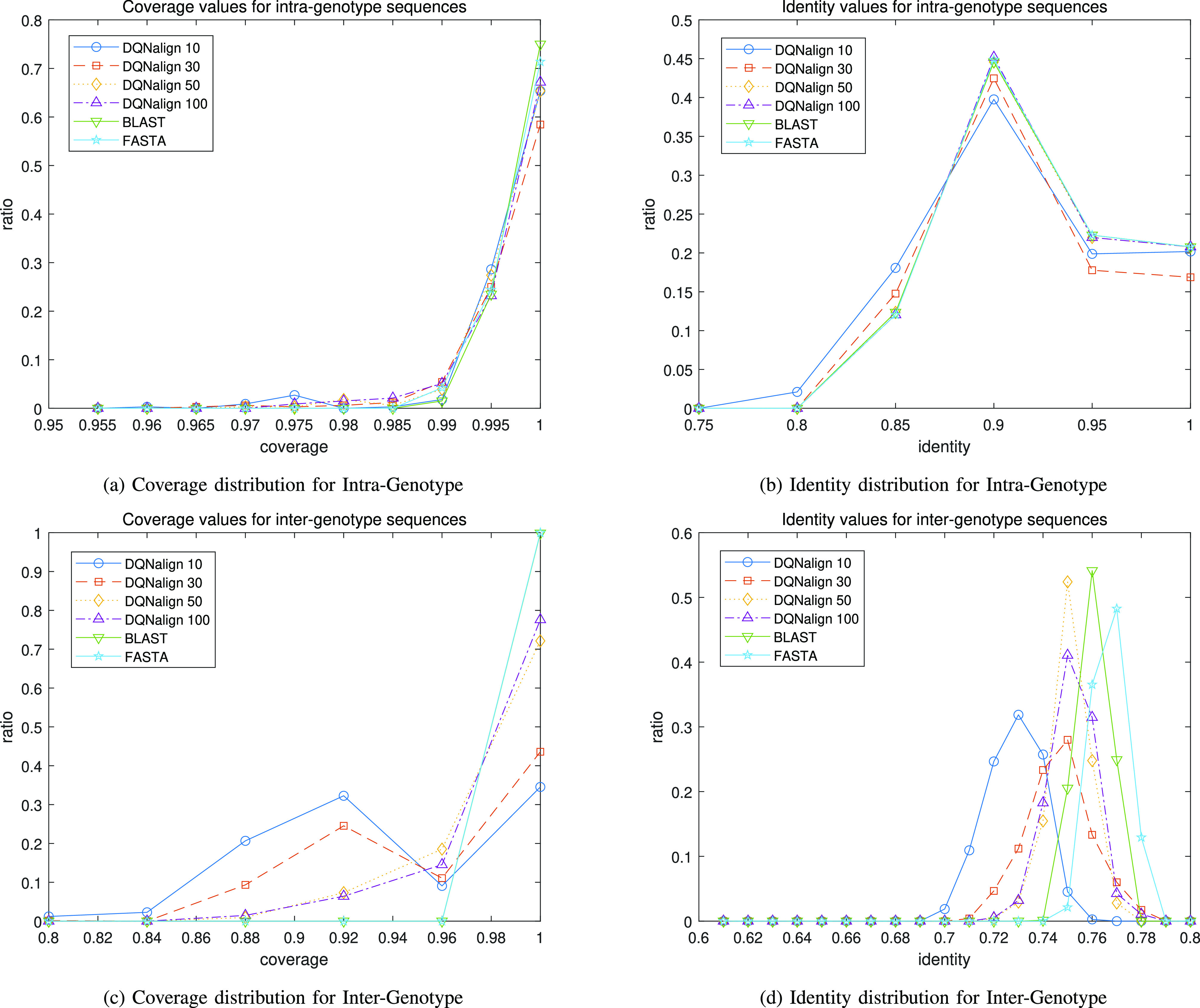
Histogram of coverage and identity values for HEV sequences: The results of the proposed DQN x-drop algorithm with several window sizes (10, 30, 50, 100) and the conventional local alignment algorithms are compared. As the window size increases, it can be observed that the performance of the proposed algorithm is getting close to that of the conventional local alignment algorithms.

## III. Results and Discussion

To show the feasibility and performance of the proposed DQN x-drop algorithm, we designed the following three comparisons: 1. Complexity analysis, 2. Performance comparison according to various window sizes, 3. Alignment time comparison according to x-drop parameter and alignment length. Based on these metrics, we will show how the DQNalign method can improve the conventional greedy x-drop algorithm. Also, all of these alignment results are included in Supplementary material S2.

### A. Complexity analysis

For complexity analysis of the proposed method, first of all, we tried to derive the relationship between the window size and the x-drop parameter. Then, we used reference papers [18], [27] to analyze the step error probability of the local best path selection method. In [18], the step error probability problem of the DQNalign was solved and summarized as the following equation based on the Gumbel distribution [24], [25].

\begin{align*}
P_{e,total} \simeq &({p}_{indel} K(e^{\lambda {score}_{gap}}+\frac{1}{4}e^{\lambda {score}_{match}}\\
& +\frac{3}{4}e^{\lambda {score}_{mismatch}})\\
&+ 2{p}_{SNP}Ke^{\lambda {score}_{gap}}) \frac{W^{2}}{e^{\lambda W {score}_{avg}}} \to 0 \tag{1}
\end{align*}

Here, $P_{e,total}$ is the step error probability to be calculated, and is expressed using the parameters of Gumbel distribution $K$ and $\lambda$. In addition, ${p}_{indel}$ and ${p}_{SNP}$ means to the probability of occurrence of indels and SNPs according to the model of evolution, and ${score}_{match}$, ${score }_{mismatch}$, ${score}_{gap}$, and ${score}_{avg}$ mean the match, mismatch, and gap scores of alignment and the average score of the sequence pair, respectively. Moreover, $W$ means the window size. From (1), we can see that this step error probability converges to zero when the window size becomes large enough.

Secondly, we intend to get the expected value of $L$ that can be obtained for a given x-drop parameter in the proposed algorithm. It is assumed that indel has the distribution of the Zipfian distribution expressed as following equation [27].

\begin{equation*}
p(l) = \frac{1/{l}^{s}}{\sum _{n=1}^{\infty }{1/{n}^{s}}} \tag{2}
\end{equation*}

In the x-drop algorithm of the proposed scheme, since the alignment is unconditionally terminated when there are $\lceil {X/{{score}_{gap}}}\rceil$ indels, the alignment length $L(X)$ is shorter than the expected length in case that indels larger than $\lceil {X/{{score}_{gap}}}\rceil$ will occur. Then, for convenience of expression, the formula $\lceil {X/{{score}_{gap}}}\rceil$ is expressed as $X_g$ as follows.

\begin{equation*}
L(X) \!<\! E[L(l>=X_g)] \!= \!\frac{1}{P(l>=X_g)} \!=\! \frac{1}{{P}_{indel} \frac{\sum _{n=X_g}^{\infty }{1/{n}^{s}}}{\sum _{n=1}^{\infty }{1/{n}^{s}}}} \tag{3}
\end{equation*}

Here, (3) can be expressed as following using the fact that $\sum _{n=X_g}^{\infty }{1/{n}^{s}}> \frac{1}{{X_g}^{s}}$.

\begin{equation*}
L(X) < \frac{1}{{P}_{indel} \frac{\sum _{n=X_g}^{\infty }{1/{n}^{s}}}{\sum _{n=1}^{\infty }{1/{n}^{s}}}} < \frac{{X_g}^s\zeta (s)}{{P}_{indel}} \tag{4}
\end{equation*}

where $\zeta (s) = \sum _{n=1}^{\infty }{1/{n}^{s}}$.

Thirdly, using this function $L(X)$, we can express the total error probability, $P_{e}$ as follows.

\begin{equation*}
\begin{aligned} P_{e} \!=\! 1-(1-P_{e,total})^{L(X)} \sim L(X)P_{e,total} < \frac{{X_g}^s\zeta (s)}{{P}_{indel}}\frac{AW^2}{e^{BW}} \end{aligned} \tag{5}
\end{equation*}

Here, we express the terms except the window size as constants A and B for the convenience of expression. In case of any $k$ less than 1, consider W that satisfies $X_g^s = e^{kBW}$. Then, (5) can be rewritten as

\begin{equation*}
\begin{aligned} P_{e} & < \lim _{W \to \infty }\frac{{X_g}^s\zeta (s)}{{P}_{indel}}\frac{AW^2}{e^{BW}} < \lim _{W \to \infty }\frac{\zeta (s)}{{P}_{indel}}\frac{AW^2}{e^{(1-k)BW}} \to 0 \end{aligned} \tag{6}
\end{equation*}

Fourthly, we will derive the relationship between window size and X parameter. According to the Lopital's theorem, if the denominator in (6) has a larger dimension than the numerator in (6), $P_{e}$ will converge to zero in case of a sufficiently large window size and $X$. From (6), we can see that for all constants $k$ less than 1, $P_e$ converges to 0 when $X_g^s < e^{BW}$ is satisfied. In case of infinitely large $X$ and $W$, it is confirmed that $P_e$ converges to 0 when $W> \frac{s}{B} \text{ ln } X_g$ is satisfied. Accordingly, in case that $P_e$ converges to 0, using $W> \frac{s}{B} \text{ ln } X_g$, we can say that the relationship between the window size and the x-drop parameter is given as follows.

\begin{equation*}
\begin{aligned} W = \frac{s}{B}\text{ ln }X_g + \alpha = \frac{s}{B} \text{ ln } X -\frac{s}{B}\text{ ln }({score}_{gap}) + \alpha \end{aligned} \tag{7}
\end{equation*}

where $\alpha > 0$.

From (7), we can see that the window size is proportional to $\text{ ln }X$ as follows.

\begin{equation*}
\begin{aligned} W \propto \text{ ln }X \end{aligned} \tag{8}
\end{equation*}

Fifthly, using the relationship between the window size and $X$ parameter in (8), we want to get the Big-O notation of computational complexity of the proposed DQN x-drop algorithm. Consider the number of alignment step shown in Fig. 2. From this figure, we can see that the proposed algorithm has a number of alignment steps proportional to $L+2X$. However, in the proposed algorithm, the deep neural network operations have $O(W)$ complexity for each alignment step. Thus, the total complexity is given in the form of $O(LW+2XW)$, which is directly proportional to the $X$ parameter. Furthermore, since the window size is proportional to $\text{ ln }X$ from (8), the proposed algorithm has the complexity of $O(LlnX + 2XlnX)$. On the other hand, from Fig. 2, we can see that the conventional greedy x-drop algorithm has a number of alignment steps proportional to $XL+X^2$ for the alignment length $L$ and the $X$ parameter. Therefore, the proposed algorithm can be said to have an advantage in view of complexity in the case of a large $X$ parameter by comparing the complexity $O(LlnX + 2XlnX)$ of proposed algorithm with the complexity $O(LX + X^2)$ of conventional algorithm.

**Fig. 4.**
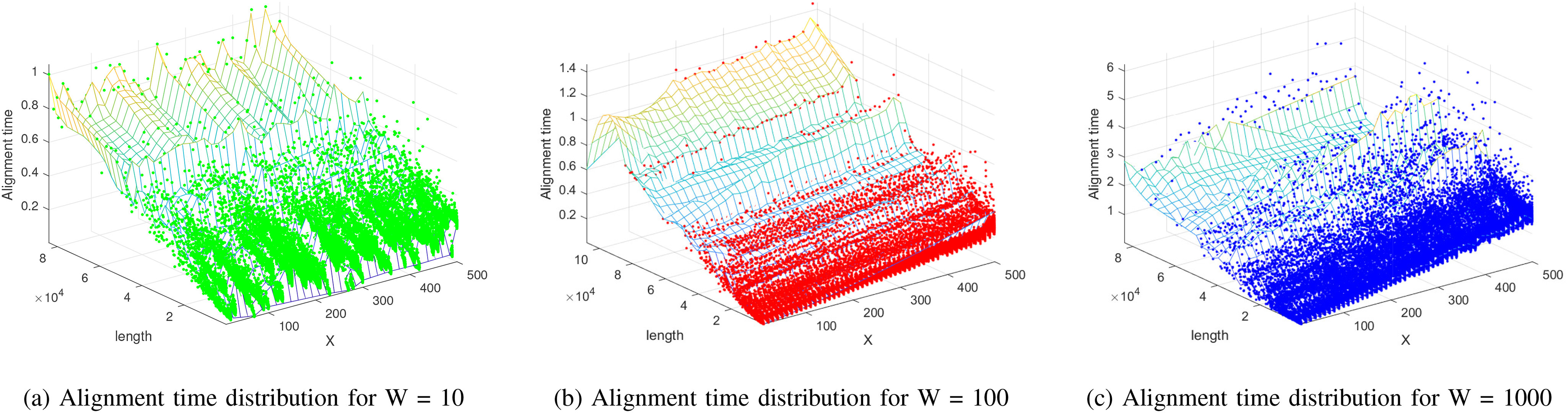
Alignment time distribution according to various X-drop parameters and alignment lengths.

### B. Performance comparison according to various window sizes

To show the performance difference according to various window sizes, we used the HEV sequence set in Table S5. We grouped HEV sequences as Intra-genotype and Inter-genotype to distinguish the performance of same genotypes from that of different genotypes, respectively. In detail, the total number of sequence pairs for all 47 sequences is 1081. Then, Intra-genotype consists of 332 sequence pairs and Inter-genotype consists of 749 sequence pairs. Also, we compared the coverage and identity performance of the conventional local alignment algorithms with those of the proposed DQN x-drop algorithm, as shown in Fig. 3. The scoring parameters and x-drop parameters were fairly set as (1,-1,-2) and 100, respectively. Especially, FASTA algorithm is also compared with proposed DQN x-drop algorithm for HEV sequence dataset. Every other parameter in this simulation is listed in Table S1, Table S2, and Table S3.

As shown in Fig. 3, alignment performance of proposed method for various window sizes of 10, 30, 50, and 100 showed significantly different trends in Intra-genotype and Inter-genotype. First, the alignment performance of proposed method for the intra-genotype shown in Fig. 3(a) and Fig. 3(b) showed similar alignment performance compared to the conventional algorithms in case of all window sizes. However, in Inter-genotype, the difference between the proposed algorithm and the conventional algorithms began to appear as shown in Fig. 3(c) and Fig. 3(d). In the inter-genotype case, we demonstrated that the proposed DQN x-drop algorithm showed relatively low alignment performance in the case of the small window sizes. However, in case of the large window size, we could observe that the proposed DQN x-drop algorithm's accuracy gradually converges to the conventional local alignment algorithms, even the superiority of proposed DQN x-drop algorithm in view of complexity. Through this simulation, we could see that the performance of the proposed algorithm increases as the window size increases.

### C. Alignment time comparison according to x-drop parameter and alignment lengths

Furthermore, we tried to compare the execution time of the conventional greedy x-drop algorithm with that of the proposed DQN x-drop algorithm for two *E.coli* sequences (*Escherichia coli* O157 and *Escherichia coli* K-12) [29]. As shown in Fig. 4, we can obtain a 3D graph of alignment time for various x-drop parameters and alignment lengths. Like the complexity analysis results, we confirmed that the alignment time of proposed algorithm is proportional to $LW+2XW$.

As can be seen from the results, an increase in the window size generally leads to a longer operation time. In a faster DDDQN structure, we replace the convolutional layer with a separable convolutional layer. Thus, we can reduce the convolutional layer's computational complexity, which is proportional to window size. However, the fully connected layer, which has constant complexity, becomes more extensive than the convolutional layer's operation time in the small window sizes.

**Fig. 5.**
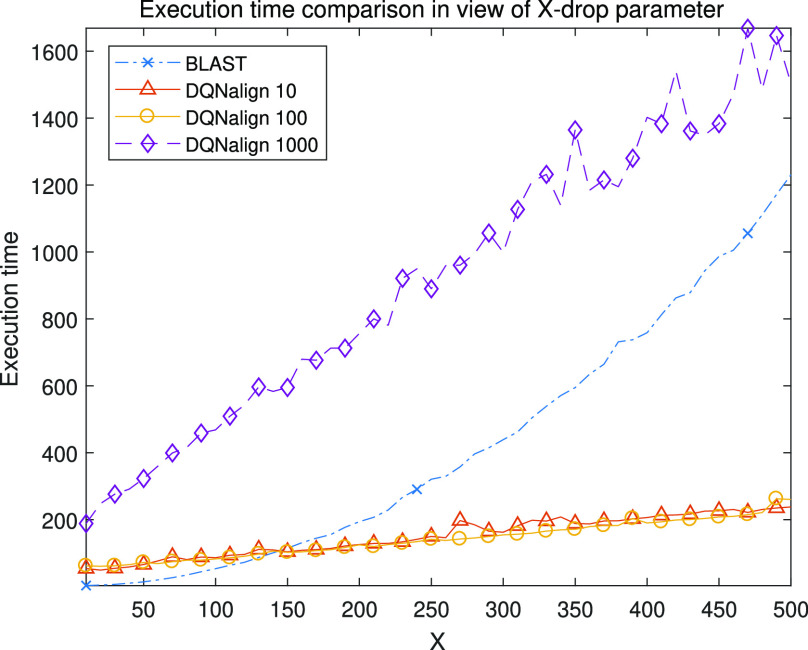
Execution time of local alignment algorithms versus various $X$ parameter

In addition, we compared the execution time of the proposed algorithm with that of the conventional algorithm, as shown in Fig. 5. We used the same scoring parameter and x-drop parameter of both methods to compare the two algorithms fairly. As a result, we confirmed that the proposed algorithm operates faster than the conventional algorithm in case of sufficiently large $X$ when the window size is 10 or 100, but when the window size is 1000, the proposed algorithm consumes more execution time than the conventional algorithm, although the proposed algorithm has linear complexity for $X$. In this case, we confirmed that the execution time results vibrate unstably when the $X$ parameter becomes large. Because the large number of candidate seeds are combined in the extension process as the X parameter increases.

In detail, when the window size is 10, the number of exact matches is decreased because the alignment performance is insufficient, which brings the increase of the total number of the network calculation. Also, the alignment execution time results are similar in case of W=10 and W=100. On the other hand, when the window size is 1000, we can confirm that the complexity increases and the computation time increase rapidly. For these reasons, we consider that 100 was the best window size for *E.coli* cases, which shows high accuracy and low complexity results as shown in Fig. 5.

## IV. Conclusion and Future Prospects

We analyzed the computational complexity of proposed and conventional algorithms theoretically, and we verified through simulation for real E.coli genomes. Further, we confirmed that the alignment time increases linearly in proportion to the X and the alignment length.

Moreover, we showed that the proposed algorithm could obtain similar alignment performance to the conventional algorithm with less complexity. That is verified by the alignment performance of the proposed algorithm for the real HEV genomes. As the window size increased, our method showed high accuracy. Despite the significant improvement in complexity, the proposed DQN x-drop method showed the same level of accuracy as the conventional method.

In addition, we compared the total execution time according to the change of X. Then, we could see that the proposed algorithm has a significant advantage in terms of complexity in the case of large X, even in the actual alignment.

A. At present, we used four small separable convolutional layers because of the complexity limitation of the current deep learning technology. But we expected that the improvement of the network operation speed, layer structure, optimizer and learning strategies can improve both the performance and complexity of the DQNalign methodology.

On the ohter hand, various MSA methodologies have been developed using dynamic programming whose complexity is proportional to the power of the number of sequences and progressive methods whose complexity is proportional to the square of the number of sequences. However, an MSA method with linear complexity has not yet been proposed. In future, we intend to develop a multiple sequence alignment methods using DQNalign. To perform neural network computation for an unspecified number of input sequences, we are willing to use recurrent neural networks used in time series data, and will show a different innovative improvement to solve the multiple sequence alignment problem.

## V. Supplementary Materials

The two supplementary materials are included in this submission. The detailed figures, simulation parameters, and the HEV sequences list are included in the Supplementary material S1. Moreover, the alignment results of the simulations are included in the Supplementary material S2.

The detailed figures, simulation parameters, and the HEV sequences list are included in the Supplementary material S1.

The alignment results of the simulations are included in the Supplementary material S2.

## References

[1] S. B. Needleman and C. D. Wunsch, “A general method applicable to the search for similarities in the amino acid sequence of two proteins,” J. Mol. Biol., vol. 48, no. 3, pp. 443–453, Mar. 1970. 5420325 10.1016/0022-2836(70)90057-4

[2] T. F. Smith and M. S. Waterman, “Identification of common molecular subsequences,” J. Mol. Biol., vol. 147, no. 1, pp. 195–197, 1981. 7265238 10.1016/0022-2836(81)90087-5

[3] S. C. Schuster, “Next-generation sequencing transforms today's biology,” Nature Methods, vol. 5, no. 1, pp. 16–18, Dec. 2007. 18165802 10.1038/nmeth1156

[4] Z. Zhang , “A greedy algorithm for aligning DNA sequences,” J. Comput. Biol., vol. 7, no. 1/2, pp. 203–214, Feb. 2000. 10890397 10.1089/10665270050081478

[5] A. Morgulis , “A fast and symmetric DUST implementation to mask low-complexity DNA sequences,” J. Comput. Biol., vol. 13, no. 5, pp. 1028–1040, Jun. 2006. 16796549 10.1089/cmb.2006.13.1028

[6] S. F. Altschul , “Gapped BLAST and PSI-BLAST: A new generation of protein database search programs,” Nucleic Acids Res., vol. 25, no. 17, pp. 3389–3402, Sep. 1997. 9254694 10.1093/nar/25.17.3389 PMC146917

[7] M. C. Frith, M. Hamada, and P. Horton, “Parameters for accurate genome alignment,” BMC Bioinf., vol. 11, no. 80, pp. 1–14, Feb. 2010. 10.1186/1471-2105-11-80 PMC2829014 20144198

[8] C. Camacho , “BLAST+: Architecture and applications,” BMC Bioinf., vol. 10, no. 421, pp. 1–9, Dec. 2009. 10.1186/1471-2105-10-421 PMC2803857 20003500

[9] W.-C. Kim , “REMiner-II: A tool for rapid identification and configuration of repetitive element arrays from large mammalian chromosomes as a single query,” Genomics, vol. 100, no. 3, pp. 131–140, Sep. 2012. 22750555 10.1016/j.ygeno.2012.06.006 PMC3428500

[10] W. R. Pearson and D. J. Lipman, “Improved tools for biological sequence comparison,” Proc. Nat. Acad. Sci., no. 85, vol. 8, pp. 2444–2448, Apr. 1988. 10.1073/pnas.85.8.2444 PMC280013 3162770

[11] R. C. Edgar, “MUSCLE: Multiple sequence alignment with high accuracy and high throughput,” Nucleic Acids Res., vol. 32, no. 5, pp. 1792–1797, Mar. 2004. 15034147 10.1093/nar/gkh340 PMC390337

[12] K. Katoh and D. M. Standley, “MAFFT multiple sequence alignment software version 7: Improvements in performance and usability,” Mol. Biol. Evol., vol. 30, no. 4, pp. 772–780, Jan. 2013. 23329690 10.1093/molbev/mst010 PMC3603318

[13] F. Sievers , “Fast, scalable generation of high-quality protein multiple sequence alignments using Clustal Omega,” Mol. Syst. Biol., vol. 7, no. 1 pp. 539, Oct. 2011, Art. no. 539. 21988835 10.1038/msb.2011.75 PMC3261699

[14] G. Marçais , “MUMmer4: A fast and versatile genome alignment system,” PLoS Comput. Biol., vol. 14, no. 1, pp. 1–14, Jan. 2018. 10.1371/journal.pcbi.1005944 PMC5802927 29373581

[15] K.-M. Chao, W. R. Pearson, and W. Miller, “Aligning two sequences within a specified diagonal band,” Bioinformatics, vol. 8, no. 5, pp. 481–487, Oct. 1992. 10.1093/bioinformatics/8.5.481 1422882

[16] H. Li, “Aligning sequence reads, clone sequences and assembly contigs with BWA-MEM,” May 2013, *arXiv:1303.3997*.

[17] L. Wang and T. Jiang, “On the complexity of multiple sequence alignments,” J. Comput. Biol., vol. 1, no. 4, pp. 337–348, Jan. 1994. 8790475 10.1089/cmb.1994.1.337

[18] Y.-J. Song, D. J. Ji, H. Seo, G. B. Han and D. -H. Cho, “Pairwise heuristic sequence alignment algorithm based on deep reinforcement learning,” IEEE Open J. Eng. Med. Biol., vol. 1, pp. 36–43, 2021. 10.1109/OJEMB.2021.3055424 PMC8901008 35402983

[19] V. Mnih , “Playing atari with deep reinforcement learning,” Dec. 2013, *arXiv:1312.5602*.

[20] H. H. Van, A. Guez, and D. Silver, “Deep reinforcement learning with double q-learning,” in Proc. 30th AAAI Conf. Artif. Intell., vol. 1, no. 1, pp. 2094–2100, Mar. 2016.

[21] Z. Wang , “Dueling network architectures for deep reinforcement learning,” Int. Conf. Mach. Learn., vol. 48, no. 1, pp. 1995–2003, Jun. 2016.

[22] A. G. Howard , “Mobilenets: Efficient convolutional neural networks for mobile vision applications,” Apr. 2017, *arXiv:1509.06461*.

[23] C. Finn, P. Abbeel, and S. Levine, “Model-agnostic meta-learning for fast adaptation of deep networks,” Jul. 2017, *arXiv:1703.03400*.

[24] R. Mott, “Maximum-likelihood estimation of the statistical distribution of Smith-Waterman local sequence similarity scores,” Bull. Math. Biol., vol. 54, no. 1, pp. 59–75, Jan. 1992. 25665661 10.1007/BF02458620

[25] H. Pang , “Statistical distributions of optimal global alignment scores of random protein sequences,” BMC Bioinf., vol. 6, no. 1, pp. 257, Oct. 2005, Art. no. 257. 10.1186/1471-2105-6-257 PMC1276786 16225696

[26] T. H. Jukes and C. R. Cantor, “Evolution of protein molecules,” in Mammalian Protein Metabolism. vol. 3, H. N. Munro, Ed. New York, NY, USA: Academic, 1969, pp. 22–96.

[27] B. Qian and R. A. Goldstein, “Distribution of indel lengths,” Proteins: Struct., Function, Bioinf., vol. 45, no. 1, pp. 102–104, Aug. 2001. 10.1002/prot.1129 11536366

[28] J. Tang , “A novel k-word relative measure for sequence comparison,” Comput. Biol. Chem., vol. 53, no. 1, pp. 331–338, Dec. 2014. 10.1016/j.compbiolchem.2014.10.007 25462340

[29] T. Hayashi , “Complete genome sequence of enterohemorrhagic Eschelichia coli O157: H7 and genomic comparison with a laboratory strain K-12,” DNA Res., vol. 8, no. 1, pp. 11–22, Feb. 2001. 11258796 10.1093/dnares/8.1.11

[30] T. Jo , “Improving protein fold recognition by deep learning networks,” Sci. Rep., vol. 5, no. 1, pp. 1–11, Dec. 2015. 10.1038/srep17573 PMC4669437 26634993

[31] H. Fukuda and K. Tomii, “DeepECA: An end-to-end learning framework for protein contact prediction from a multiple sequence alignment,” BMC Bioinf., no. 21, vol. 1, pp. 1–15, Jan. 2020. 10.1186/s12859-019-3190-x PMC6953294 31918654

[32] S. Seo , “DeepFam: Deep learning based alignment-free method for protein family modeling and prediction,” Bioinformatics, no. 34, vol. 13, pp. i254–i262, Jul. 2018. 10.1093/bioinformatics/bty275 PMC6022622 29949966

[33] J. K. Senter , “Unaligned sequence similarity search using deep learning,” in Proc. IEEE Int. Conf. Bioinf. Biomed., Nov. 2019, pp. 1892–1899.

[34] D. Quang and X. Xie, “DanQ: A hybrid convolutional and recurrent deep neural network for quantifying the function of DNA sequences,” Nucleic Acids Res., no. 44, vol. 11, pp. e107–e107, Apr. 2016. 10.1093/nar/gkw226 PMC4914104 27084946

[35] R. Jafari, M. M. Javidi, and M. K. Rafsanjani, “Using deep reinforcement learning approach for solving the multiple sequence alignment problem,” SN Appl. Sci., no. 1, vol. 6, pp. 1–12, May 2019.

[36] M. Gao and J. Skolnick, “A novel sequence alignment algorithm based on deep learning of the protein folding code,” Bioinformatics, vol. 1, no. 1, pp. 1–7, Sep. 2020. 10.1093/bioinformatics/btaa810 PMC8599902 32960943

