# Supplementary material for: Local Alignment of DNA Sequence Based on Deep Reinforcement Learning
Source: IEEE Open J Eng Med Biol. 2021 Apr 27;2:170–8. doi: 10.1109/OJEMB.2021.3076156 (PMC8975175; doi:10.1109/OJEMB.2021.3076156)
Supplement: The detailed figures, simulation parameters, and the HEV sequences list are included in the Supplementary material S1. [file supp1-3076156.pdf]

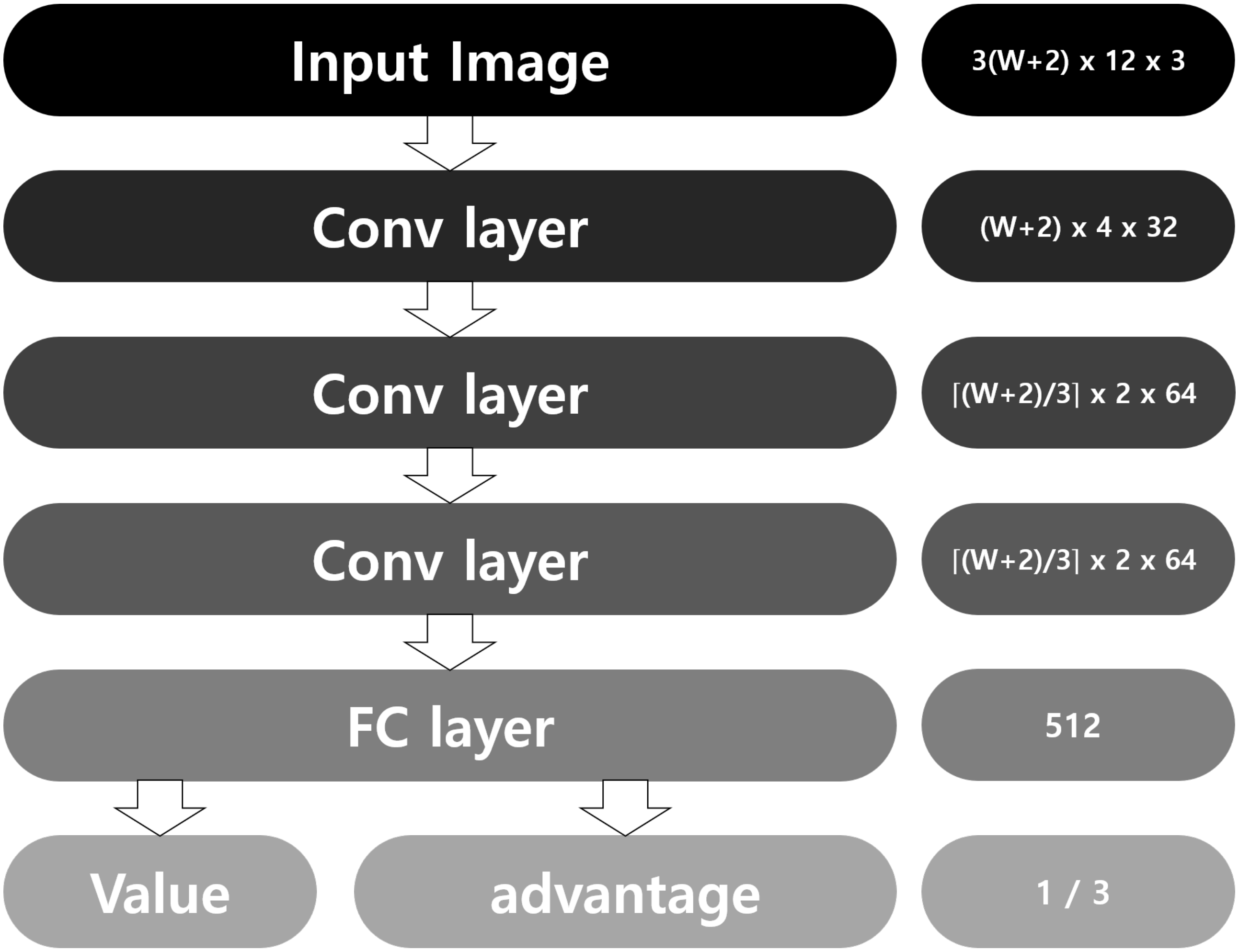

Fig. S1. Detailed network architecture of Dueling Double Deep Q network (DDDQN)

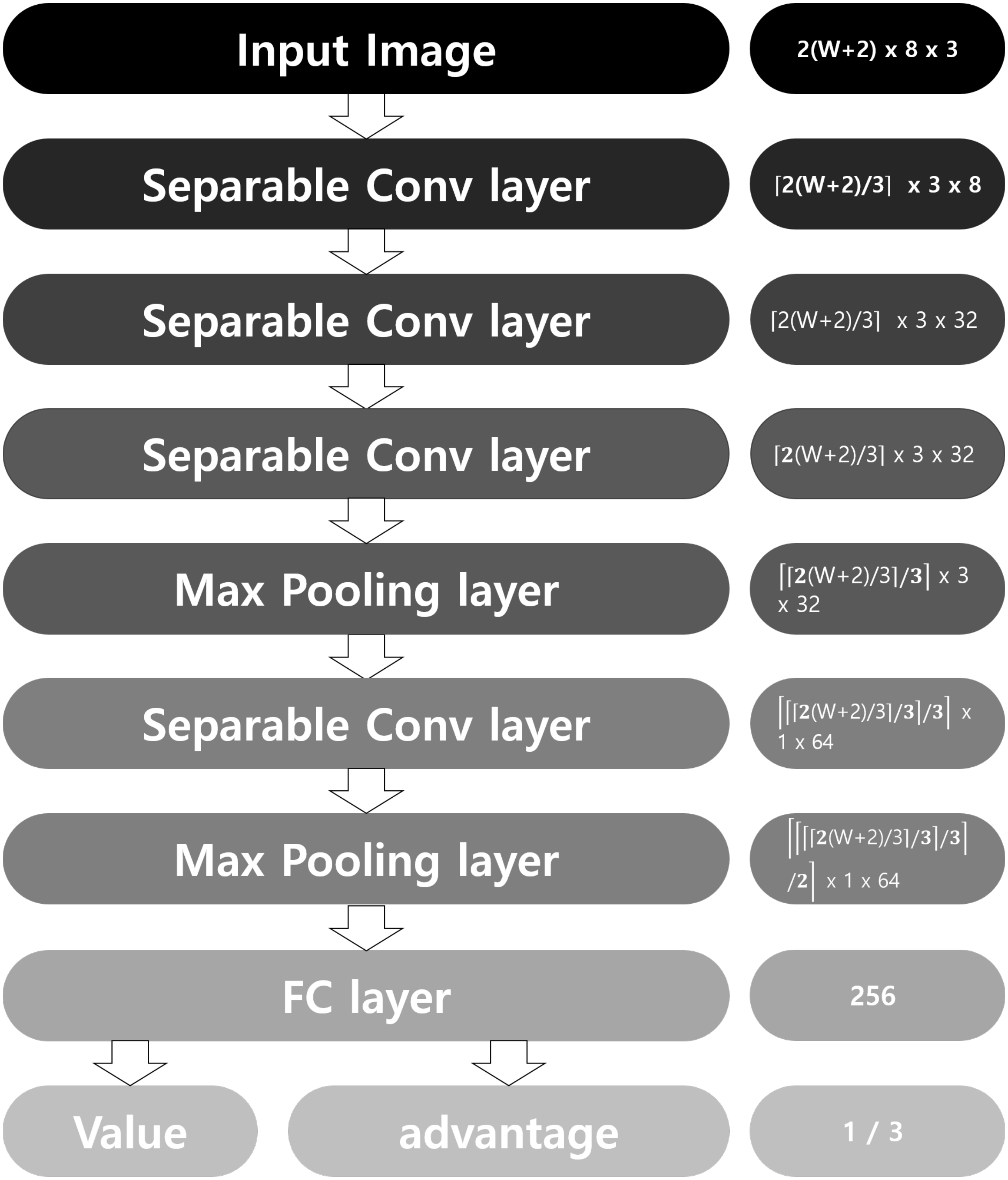

Fig. S2. Detailed network architecture of separable convolutional layer based network (faster DDDQN)

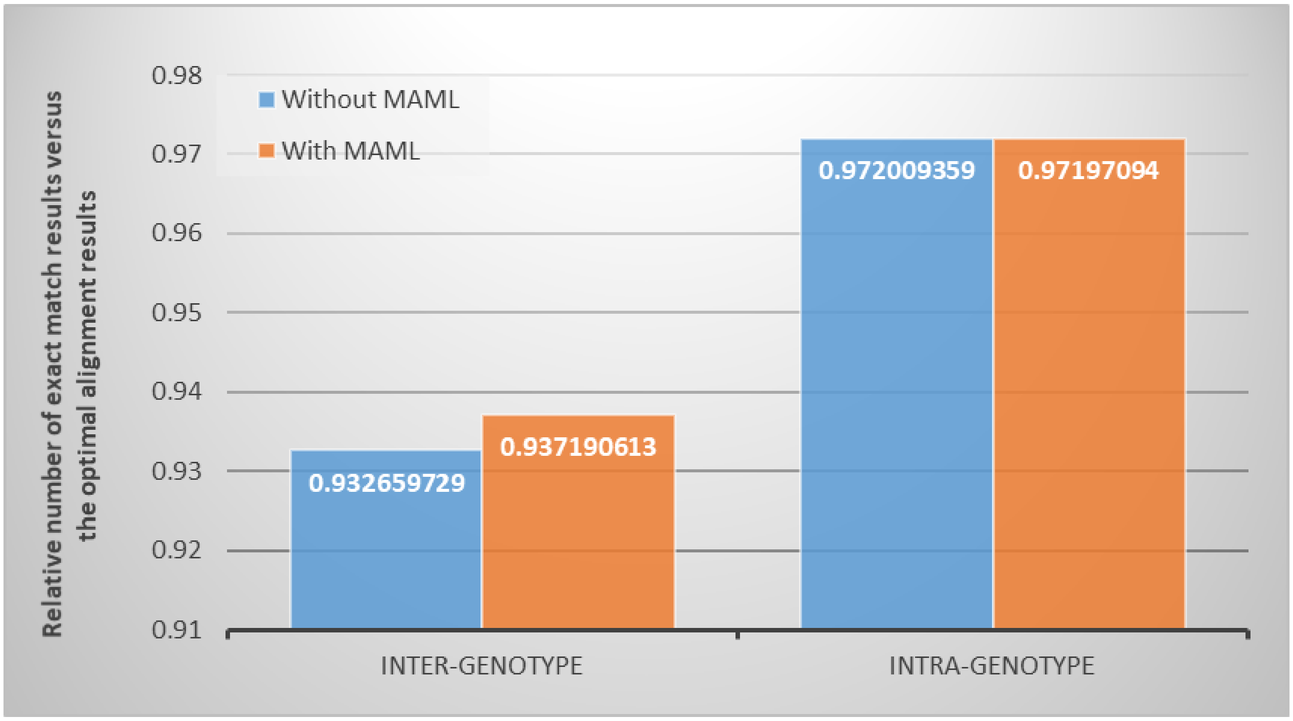

Fig. S3. The effect of meta-training procedure

TABLE S1. Parameters used in the proposed local alignment method based on DQNalign algorithm

Parameters	HEV simulation case	E.coli simulation case
Window size for filtering	20	20
Threshold for filtering	0.6	0,6
Word size	14	14
Allowable size per word	1	1
Space size	2	2
Minimum seed length	25	100
Alignment score (match/mismatch/Gap)	(1/-1/-2)	(1/-1/-2)
Score threshold (in ungapped extension)	-10	-10
Window size	10, 30, 50, 100	100, 1000
X-drop parameter	100	10, 20, ..., 500

TABLE S2. Parameters used in the BLAST software

Parameters	megablast	HEV simulation case	E.coli simulation case
Alignment score (match/mismatch)	(1/-2)	(1/-1)	(1/-1)
Gap panelty (open/extend)	(5/2)	(2/2)	(2/2)
X-drop parameter in ungapped extension	20	100	10, 20, ... , 500
X-drop parameter in gapped extension	30	100	10, 20, ... , 500
X-drop parameter in final gapped extension	100	100	10, 20, ... , 500

※ The unlisted parameters of the BLAST are the same as those of megablast.

TABLE S3. Parameters used in the FASTA software

Parameters	FASTA
Alignment score (match/mismatch)	(5/-4)
Gap panelty (open/extend)	(14/4)
KTUP (k-word)	6

TABLE S4. Parameters used in the meta-training procedure

Parameters	Small window size	Large window size
Sequence length (l)	8000	20000
Probability of SNP (p_{SNP})	0.001, 0.002, ..., 0.3	0.001, 0.002, ..., 0.3
Probability of indel (p_{indel})	0.0001, 0.0002, ..., 0.03	0.0001, 0.0002, ..., 0.03
Maximum length of indel (I_{max})	5, 6, ..., 20	5, 6, ..., 20
Zipfian distribution parameter (s)	1.6	1.6
Window size (s)	10, 30, 50, 100	1000

TABLE S5. Benchmarked real genome sequences

NO.	STRAIN NAME	ACCESSION NO.	GENOTYPE	LENGTH
1	B1	M73218	I	7207
2	B2	D10330	I	7194
3	I3	AF076239	I	7194
4	NP1	AF051830	I	7199
5	P2	AF185822	I	7143
6	Yam-67	AF459438	I	7206
7	C1	D11092	I	7207
8	C2	L25595	I	7221
9	C3	L08816	I	7176
10	C4	D11093	I	7194
11	China Hebei	M94177	I	7200
12	P1	M80581	I	7138
13	I1	X98292	I	7202
14	Morocco	AY230202	I	7212
15	T3	AY204877	I	7170
16	M1	M74506	II	7180
17	HE-JA10	AB089824	III	7262
18	JKN-Sap	AB074918	III	7256
19	JMY-HAW	AB074920	III	7240
20	SW-US1	AF082843	III	7207
21	US1	AF060668	III	7202
22	US2	AF060669	III	7277
23	JBOAR1-HYO04	AB189070	III	7247
24	JDEER-HYO03L	AB189071	III	7230
25	JJT-KAN	AB091394	III	7218
26	JIMO-HYO03L	AB189072	III	7180
27	JRA1	AP003430	III	7230
28	JSO-HYO03L	AB189073	III	7180
29	JTH-HYO03L	AB189074	III	7180
30	JYO-HYO03L	AB189075	III	7180
31	SWJ570	AB073912	III	7257
32	KYRGYZ	AF455784	III	7239
33	ARKELL	AY115488	III	7255
34	HE-JA1	AB097812	IV	7258
35	HE-JK4	AB099347	IV	7250
36	HE-JI4	AB080575	IV	7186
37	JAK-Sai	AB074915	IV	7236
38	JKK-SAP	AB074917	IV	7235
39	JSM-SAP94	AB161717	IV	7202
40	JSN-SAP-FH	AB091395	IV	7234
41	JSN-SAP-FH02C	AB200239	IV	7251
42	JTS-SAP02	AB161718	IV	7202
43	JYW-SAP02	AB161719	IV	7202
44	SWJ13-1	AB097811	IV	7258
45	SWCH25	AY594199	IV	7270
46	T1	AJ272108	IV	7232
47	CCC220	AB108537	IV	7193
