# Supplementary material for: Local Alignment of DNA Sequence Based on Deep Reinforcement Learning
Source: IEEE Open J Eng Med Biol. 2021 Apr 27;2:170–8. doi: 10.1109/OJEMB.2021.3076156 (PMC8975175; doi:10.1109/OJEMB.2021.3076156)
Supplement: The alignment results of the simulations are included in the Supplementary material S2. [file supp2-3076156.pdf]

IEEE Open Journal of
Engineering in Medicine and Biology


# Supplementary Material S2

## Local Alignment of DNA Sequence Based on Deep Reinforcement Learning

Yong-Joon Song, and Dong-Ho Cho*, *Senior Member, IEEE*

THIS Supplementary Material deals with the list of sequence alignment result files in this paper. The data is available at https://drive.google.com/file/d/1mrCpXLzQ9q2APCFNsm2ifJhXw8SDeNpe/view?usp=sharing.

TABLE A1. List of the alignment result files

| File name | Description |
| --- | --- |
| HEV_DQNalign10 | Sequence alignment results for 1081 HEV sequence pairs (W=10) |
| HEV_DQNalign30 | Sequence alignment results for 1081 HEV sequence pairs (W=30) |
| HEV_DQNalign50 | Sequence alignment results for 1081 HEV sequence pairs (W=50) |
| HEV_DQNalign100 | Sequence alignment results for 1081 HEV sequence pairs (W=100) |
| Ecoli_DQNalign100 | Sequence alignment results for *E.coli* sequence pair (W=100) |
| Ecoli_DQNalign1000 | Sequence alignment results for *E.coli* sequence pair (W=1000) |
| BLAST | Sequence alignment results for *E.coli* sequence pair |
